# First Discovery on Interspecific Parental Care of Siberian Stonechat ( *Saxicola maurus* ) Nestlings Provided by a Male White Wagtail ( *Motacilla alba* )

**DOI:** 10.1002/ece3.71188

**Published:** 2025-04-02

**Authors:** Jun‐hui Zheng, Kai‐xing Dai, Zheng‐wang Zhang, Fen‐liang Kuang, De‐pin Li

**Affiliations:** ^1^ Institute of Eastern‐Himalaya Biodiversity Research Dali University Dali Yunnan China; ^2^ Key Laboratory for Biodiversity Science and Ecological Engineering, College of Life Sciences Beijing Normal University Beijing China; ^3^ School of Chemistry and Environment Yunnan Minzu University Kunming China

**Keywords:** interspecific feeding, nest sanitation, Siberian stonechat, white wagtail

## Abstract

Interspecific parental behavior has been reported worldwide, particularly in Europe and North America, yet relatively little information is available from East Asia. We present the first known instance of interspecific parental care by a white wagtail ( *Motacilla alba* ) at a Siberian stonechat ( *Saxicola maurus* ) nest, documented via video footage in the farmland of Northwest Yunnan, China. We quantify parental behaviors and describe aggressive interactions between the adult Siberian stonechats and the white wagtail. The white wagtail lightened the provisioning load of the male and female stonechats and increased total provisioning to nestlings to some extent. Moreover, the investment of the three birds, in terms of food delivery and fecal‐sac removal, was evenly distributed among the five nestlings at the nest. Although proximate causes of this behavior are unclear, close nest vicinity with the host young's loud begging and the loss of the wagtail's offspring or eggs may have triggered this behavior.

## Graphical abstract

We present the first known interspecific parental care by a white wagtail ( *Motacilla alba* ) at a nest of Siberian stonechat ( *Saxicola maurus* ), documented by video in the farmland of northwest Yunnan, China. The White wagtail did significantly more feeding attempts than either parent of the Siberian stonechats but did not show a significant difference in sanitation maneuvers compared to each sex of the stonechat parent. Moreover, the three birds' investment, in terms of food delivery and fecal‐sac removal, was distributed evenly among the five nestlings at the nest.

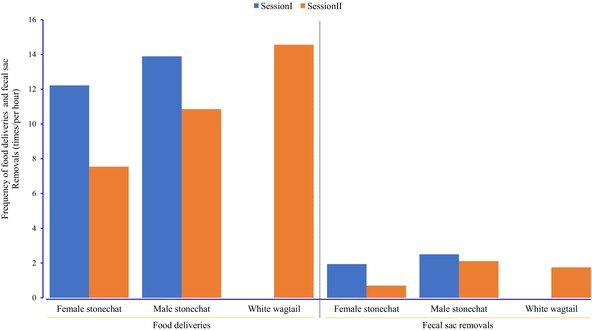

## 1 Introduction

Interspecific parental care in birds is an uncommon behavior, in which an individual (mostly offspring) of one species provides food to nestlings of another species and/or helps with the removal of fecal sacs (Halley and Heckscher 2013; Janus and Gow 2021). Although the occurrences of interspecific parental care are rare outside the context of brood parasitism (Krištín et al. 2009), interspecific feeding has been documented in 107 “helper” species (i.e., the species exhibiting interspecific feeding) from 41 families across 10 orders (Harmáčková 2021). Concerning interspecific parental care, Shy (1982) proposed eight categories of possible causes for this behavior: (I) the presence of mixed clutches in one nest, (II) the diversion of a bird's attention to other nests due to losing its clutch, which was physiologically in a parenting state, (III) the adult birds are often attracted by the neighboring nest and exhibit parental care behavior toward the nestlings of theses nests, (IV) the individuals redirect their attention to other nests due to an inability to find a mate, (V) birds were stimulated by the loud begging calls from another species nestlings, (VI) birds adopted the orphaned progeny of another species, (VII) the male intended to feed the nestlings of other species while its mate incubates eggs, and (VIII) other miscellaneous causes.

Whether interspecific care is an adaptive behavior of the helper remains debatable because it does not provide either direct or indirect fitness benefits (Brown 1983; Brown 1987; Halley and Heckscher 2013). Others have suggested that this behavior may offer potential benefits for helpers for acquiring parental skills that could be useful in the future when caring for their own offspring (Riedman 1982), or as a consequence of normal adaptive sexual solicitation by the recipient species (Halley and Heckscher 2013). However, the reproductive benefits for the heterospecific helper and the significance of this behavior in an evolutionary context remain unclear (Shy 1982; Harmáčková 2021). Detailed records of interspecific parental care can enhance our overall understanding of the evolution of this behavior (Liu et al. 2024), and it is crucial to provide comprehensive descriptions of the conditions underlying helper‐host interactions (Krištín et al. 2009; Harmáčková 2021).

To date, cases of interspecific feeding have been documented globally excluding Africa, with the majority recorded in Europe and North America, regions with a long ornithological and research tradition (Harmáčková 2021). In China, only four cases of interspecific parental care have been reported (excluding interspecific brood parasitism; Jiang et al. 2016; Luo et al. 2018; Liu et al. 2024; Lu et al. 2024), despite harboring more than 10% of the global bird species (Zheng 2023). Here, we quantify interspecific parental care at a nest of Siberian stonechats ( *Saxicola maurus* , hereafter stonechat) in Northwest Yunnan, China, detected using a video camera. The nest was attended by two adult stonechats and an adult male white wagtail ( *Motacilla alba* , hereafter referred to as wagtail). The number of food deliveries and fecal sac removals was compared between the stonechats and the wagtail. We also tested whether their investments were randomly distributed among the nestlings and described aggressive interactions that occurred between stonechats and the wagtail. Finally, we discuss the proximate reasons summarized by Shy (1982) to explain interspecific nestling care.

## 2 Methods

The observations described herein occurred between March and July 2021 during a study on the breeding ecology of stonechats and wagtails at a long‐term study site located in a farmland environment (25°29′15″~25°57′54″ N, 99°55′52″~99°58′54″ E), near Fengyu Town of Eryuan County, Northwest Yunnan, China. The study site is a flat area consisting of arable fields bordered by semi‐natural grass vegetation dominated by native plant species, such as feather finger grass ( *Chloris virgata* ), branched horsetail ( *Equisetum ramosissimum* ), annual bluegrass ( *Poa annua* ) and *Artemisia* Sp. The main crops cultivated in the arable fields during data collection were corn ( *Zea mays* ), tobacco ( *Nicotiana tabacum* ) and barley (*Hordeum* Sp.).

Stonechats and wagtails are the common species that breed in our study area. Their nests were usually constructed on the ground with dense grass of field boundaries or old fallows in farmland environment (Li 2020; Pers. Observ.). The nest of interest was discovered on May 5, 2021, during a search along field boundaries, and subsequently monitored by inspecting the nest once every 4 days until May 16, when all five offspring had fledged. When the offspring were 9 days old (hatch day = day 1), we positioned a video camera (Sony HDR‐750E) to record the feeding behavior of the stonechat. The recording time took place from 8:18 AM to 5:40 PM on 12 May. For individual identification during videotaping, the forehead and bill of each offspring within a brood were marked using non‐toxic colored pen, with one or combination of the following basic colors: red, green, purple, and no color (i.e., non‐color).

In the laboratory, video footage was reviewed using Windows Movie Maker software (version 6.0). The whole video footage was divided into two sessions: one before and one after the wagtail initially began caring for the stonechat nestlings, yielding 3.6 h and 5.7 h of video footage for analysis, respectively. For each session, parental contributions to individual nestlings were recorded. We also quantified (1) the number of food deliveries to each nestling and (2) the number of fecal sacs removed from each nestling. Chi‐squared tests were used to compare the differences in the investments of the three adults in the delivery of food and fecal sac removal, as well as whether their investments were randomly distributed among each nestling. Data processing and statistical analyses were conducted with R3.6.1 (R Core Team 2019).

## 3 Results

**FIGURE 1:**
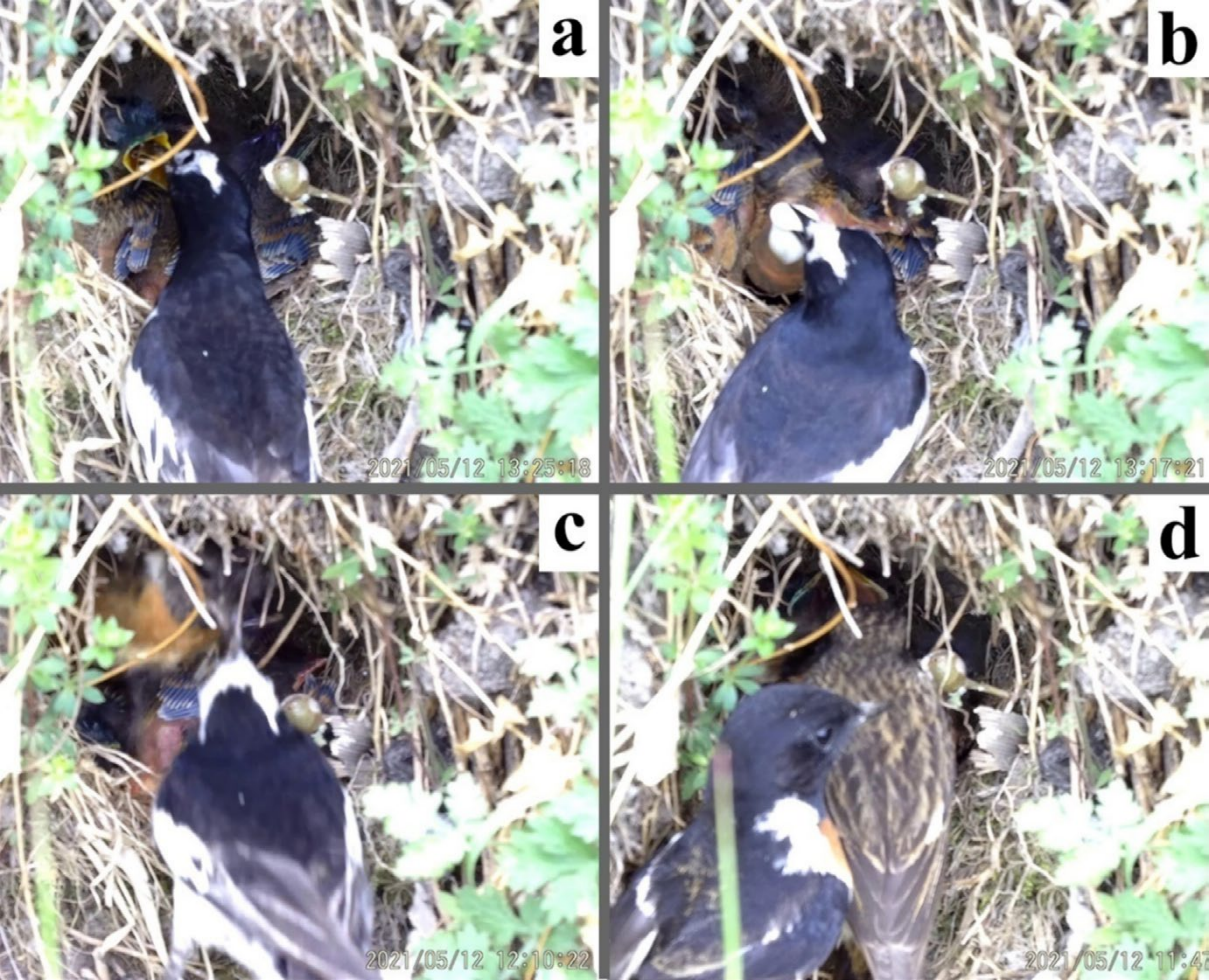
Still images from a video recording of a Siberian stonechat ( *Saxicola maurus* ) nest in Eryuan County attended by an adult male white wagtail ( *Motacilla alba* ): (a) The white wagtail feeding nestlings, (b) the white wagtail removing a fecal sac from the nest, (c) female Siberian stonechat and the white wagtail attacking each other at the nest, and (d) the female Siberian stonechat feeding nestling and the male stonechat at the edge of the nest just after completing a food delivery.

The wagtail first appeared at the nest after 2.9 h of video recording. After visiting the nest three times without food, it returned to feed one of the nestlings and then immediately flew away. Then, the female stonechat returned to the nest within 10 s of the wagtail's departure to feed one of the nestlings, jumped into the nest, and guarded it, possibly indicating awareness of the helper's visit. After 13 s, the wagtail returned to the nest without food and aggressively attacked the female stonechat hiding in the nest with its bill for several times. The female stonechat counter‐attacked and evicted the wagtail away from the nest (Figure 1c, Video S1). In one recorded interaction, the male stonechat also attacked and chased the wagtail when the latter came back to feed nestlings at 6.6 h into the video (Video S2). After 36 min, the wagtail remained in the area nearing the nest after feeding the nestlings and exhibited aggressive behavior toward the male stonechat when it approached the nest to feed the offspring (Video S3). Thereafter, the two adult stonechats and the wagtail continued to provide food and remove the fecal sacs for nestlings without attacking each other during the video recording.

**FIGURE 2:**
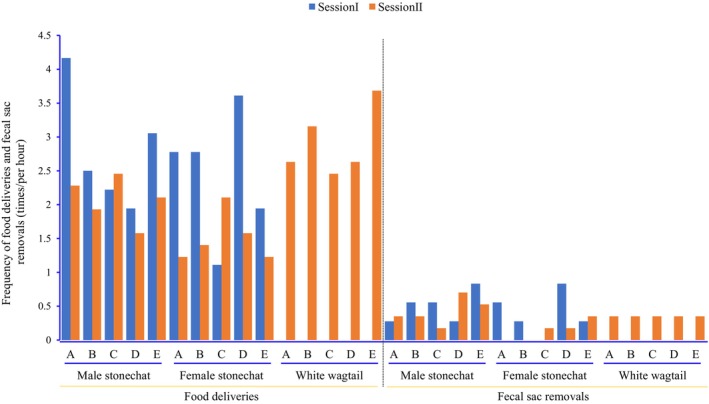
Differential food deliveries and fecal sac removals to five offspring (A–E) by two adult Siberian stonechats and an adult white wagtail at a Siberian stonechat nest in Eryuan County. Session I and session II refer to the periods before and after the wagtail began participating in the care of the stonechat nestlings.

During video session I (3 h and 36 min), which was before the wagtail took care of stonechat nestlings, the stonechats delivered food 94 times: 44 of which were by the female and 50 times by the male stonechat (*χ* ^2^ = 0.383, df = 1, *p* = 0.536). No individual nestling was favored (*χ* ^2^ = 4.526, df = 4, *p* = 0.339) (Figure 2). The female and male stonechat removed fecal sacs 7 times and 10 times, respectively (*χ* ^2^ = 0.529, df = 1, *p* = 0.467), and did not favor any individual nestling (*χ* ^2^ = 0.941, df = 4, *p* = 0.919) (Figure 2). During video session II (5 h and 42 min), which was after the wagtail began caring for nestlings, the wagtail helper delivered food to the nestlings 83 times, compared to 49 and 55 deliveries by the female and male stonechats, respectively (*χ* ^2^ = 10.567, df = 2, *p* = 0.005). Food deliveries by the three adults were evenly distributed among the five stonechat offspring (*χ* ^2^ = 1.204, df = 4, *p* = 0.887) (Figure 2). The male wagtail, female stonechat, and male stonechat removal of fecal sacs was 10, 4, and 12 times, respectively (*χ* ^2^ = 4.00, df = 2, *p* = 0.135), and no individual nestling was favored (*χ* ^2^ = 3.615, df = 4, *p* = 0.461) (Figure 2).

**FIGURE 3:**
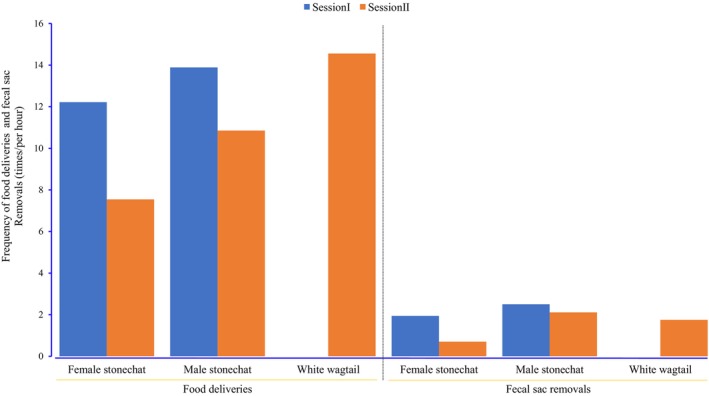
Frequency of food deliveries and fecal sac removals at the nest of a Siberian stonechat attended by two adult Siberian stonechats and an adult White wagtail in Eryuan County. Session I and session II refer to the periods before and after the wagtail began caring for the stonechat nestlings.

The total provisioning rate was 26.11 times per hour during session I, compared to 32.46 times per hour during session II, with additional care by wagtail helper (*χ*2 = 1.712, df = 1, *p* = 0.191). The provisioning rate by the female stonechat appeared to decline by approximately half, with 7.54 times per hour during session II, compared to 12.22 times per hour during session I (Figure 3). However, the difference was not statistically significant (*χ* ^2^ = 1.108, df = 1, *p* = 0.292). Meanwhile, the provisioning rate of the male stonechat was 13.89 times per hour during session I and 10.35 times per hour during session II, respectively (*χ* ^2^ = 0.517, df = 1, *p* = 0.472; Figure 3). The total fecal sac removal rate was similar between the two sessions (session I: 4.44 times per hour, session II: 4.56 times per hour; *χ* ^2^ = 0.002, df = 1, *p* = 0.968). The rate of fecal sac removals by the female stonechat during session I (1.94 times per hour) was more than twice as high as its rate during session II (0.70 times per hour), although the difference was not statistically significant (*χ* ^2^ = 0.582, df = 1, *p* = 0.445; Figure 3). In contrast, the male stonechat removed fecal sacs at similar rates between the two sessions (session I: 2.50 times per hour, session II: 2.11 times per hour; *χ* ^2^ = 0.002, df = 1, *p* = 0.968; Figure 3).

## 4 Discussion

Parental care provided by the wagtail exceeded that of both stonechats, especially in feeding attempts for nestlings, as the wagtail delivered significantly more food than either stonechat. This is similar to a finding in veery ( *Catharus fuscescens* ) nestlings, which were provided with food more frequently by a wood thrush ( *Hylocichla mustelina* ) helper (Halley and Heckscher 2013). It is common for the heterospecific nest helper to contribute to nest sanitation (Schaeffer et al. 2009; Luo et al. 2018; Liu et al. 2024), often to a greater degree than the host parents (Halley and Heckscher 2013). In our observation, the fecal‐sac removals by the wagtail were not significantly different from those of either the male or female stonechat. In one similar study, food deliveries by the helper ( *Hylocichla mustelina* ) and two adult hosts ( *Catharus fuscescens* ) were not distributed evenly among the offspring, with the helper and female host tending to provide more food deliveries to the smaller size nestling (Halley and Heckscher 2013). Although there were size disparities among siblings with body mass ranging from 12.85 ~ 15.90 g (mean weight of 8‐day‐olds =14.31 ± 1.10 g; *n* = 5) in the stonechat nest, no nestling was preferentially fed by both hosts and helper. This suggests that offspring size may not be a cue used by either stonechat parents or wagtail helper to allocate their investment.

In addition, compared with the session when the wagtail helper cared for the nestlings, the total provisioning rate per hour increased, whereas each sex of stonechat reduced its provisioning rate after the additional care by the wagtail helper (Figure 3), although the difference was not statistically significant between the two sessions. These results suggest that total provisioning and load‐lightening increased with additive feeding investment by the wagtail helper to some extent, possibly leading to direct fitness benefits for the stonechat parents, as documented in some studies referring to contributions by intraspecific helpers in cooperative breeding systems (Hatchwell et al. 2004; Van Boheemen et al. 2019). It is difficult to disentangle the effect of the wagtail helper on increasing the success of the current reproductive attempt and/or improving the survival and future reproductive output of the stonechat parents due to the limited sample size in this study. Hopefully, more detailed observations are expected in the future due to the development of monitoring devices (Luo et al. 2018; Harmáčková 2021), which could help to disentangle the current and/or future fitness benefits for parents provided by the interspecific helper.

Several factors may have contributed to this uncommon instance of interspecific parental care (Shy 1982). Our focal nest only contained stonechat nestlings, as indicated by the typical feathering of the 9‐day‐old nestlings. Therefore, the wagtail's activity at the nest is not explained by the presence of its nestlings in the nest (no mixed brood). Yet, a mixed brood including a wagtail has been reported, where the wagtail also cared for the nestlings (with European robins, Lack 1953).

Among the possible factors, loud begging calls from nestlings in a nearby nest may be the most important trigger for interspecific activities (Harmáčková 2021). In our study area, stonechats and wagtails not only share similarities in nest habitat, but also have similarly shaped nests; both build open cup nests on the ground of field boundaries or old fallows in farmland environments. Three wagtail nests were found near the stonechat nest, at a distance of 100~200 m. On the day when interspecific parental care was recorded, the nestlings of the nearest wagtail nest were 4 days old, and finally fledged, while the other two nests experienced reproductive failures after approximately 5 days and 20 days, respectively. Unfortunately, we were unable to identify the adult wagtail individuals because none were banded, and therefore, we could not determine from which nest the helper originated. We speculate that the wagtail helper from the nearest nest may have been attracted by the loud begging of the stonechat nestlings nearby, but the possibility of other wagtail helpers that lost their own clutch cannot be excluded. The fact that the level of prolactin, a hormone involved in parental behavior (Buntin 1996), does not decrease immediately after nest failure might have caused the wagtail to treat the stonechat chicks as its own. This has been suggested as an explanation for nest usurpation by an eared dove ( *Zenaida auriculata* ) of the nestlings of the creamy‐bellied thrush ( *Turdus amaurochalinus* ) (Segura et al. 2016). Additionally, emperor penguins (*Aptenodytes fosteri*) that have lost their own offspring may attempt to kidnap chicks, but this behavior ceases once the hormone prolactin is blocked (Angelier et al. 2006).

The defensive behavior exhibited by the stonechats against the male wagtail did not deter the wagtail helper from caring for its offspring. This has also been documented in other cases of interspecific parental care (Batisteli and Sarmento 2016; Janus and Gow 2021). Furthermore, only the male Wagtail was caring for nestlings in the alien nest, which may support the hypothesis that males are more frequently helpers than females, perhaps because they have more time and opportunities (Skutch 1961; Cockburn 1998).

To the best of our knowledge, this is the first report of an adult wagtail feeding nestlings and removing fecal sacs from a stonechat nest. Stonechats and wagtails share similar nest habitats and overlap in their breeding periods in our study area, where the frequent agricultural interference during the breeding season could affect the suitability of nesting habitat for both species. We also observed several cases in which wagtails took over failed stonechat nests in early stages, such as the incubation period or in the brooding period when nestlings were less than 3 days old (unpublished data), indicating potential competition for nest sites. Our opportunistic findings of interspecific parental care provide valuable insights for future studies on the reproductive ecology of these two bird species, which may directly compete for nest sites in human‐dominated habitats.

## Author Contributions

**Jun‐hui Zheng:** conceptualization (equal), data curation (lead), investigation (supporting), writing – original draft (equal), writing – review and editing (supporting). **Kai‐xing Dai:** conceptualization (equal), data curation (supporting), investigation (lead), writing – original draft (equal). **Zheng‐wang Zhang:** project administration (supporting), writing – review and editing (supporting). **Fen‐liang Kuang:** formal analysis (supporting), visualization (supporting). **De‐pin Li:** conceptualization (lead), formal analysis (lead), project administration (lead), writing – original draft (lead), writing – review and editing (lead).

## Conflicts of Interest

The authors declare no conflicts of interest.

## Supporting information


Videos S1‐S3.

## Data Availability

The data (Videos S1–S3) supporting this study's findings are available in Dryad at http://datadryad.org/stash/share/oC3ZfqWg_Qcd6Z9tBuOTQoHV4wO‐nB7GNxyal‐3uABA.
